# The infrastructure of electrophysiology centers impacts the management of cardiac tamponade—Results from a national survey

**DOI:** 10.1002/clc.24096

**Published:** 2023-08-01

**Authors:** Laura Rottner, Stefan Reubold, Sophie Schönhofer, Bruno Reißmann, Feifan Ouyang, Julius Obergassel, Ilaria My, Fabian Moser, Jan‐Per Wenzel, Marc Lemoine, Daniel Steven, Philipp Sommer, Paulus Kirchhof, Andreas Rillig, Andreas Metzner

**Affiliations:** ^1^ Department of Cardiology University Heart and Vascular Center Hamburg Hamburg Germany; ^2^ Asklepios Klinik Harburg Hamburg Germany; ^3^ University Heart Center, University of Cologne Cologne Germany; ^4^ Herz‐ und Diabeteszentrum NRW, Bad Oeynhausen, Ruhr‐University of Bochum Bad Oeynhausen Germany; ^5^ DZHK, partner site Hamburg/Kiel/Lübeck Hamburg Germany; ^6^ Institute of Cardiovascular Sciences University of Birmingham Birmingham UK

**Keywords:** cardiac tamponade, catheter ablation, institutional infrastructure, survey

## Abstract

**Background:**

Although electrophysiological (EP) centers have institutional standards, evidence on management of cardiac tamponade is lacking.

**Aim and Methods:**

A physician‐based survey was conducted by sending out questionnaires to all hospitals in Germany performing EP procedures. To evaluate the infrastructure of EP centers and the impact of center volume and onsite cardiac surgery on the management of cardiac tamponade, the results of the survey were analyzed for low‐volume (0–250 procedures per year), mid‐volume (250–500 procedures), and high‐volume (>500 procedures) centers, as well as for centers with and without onsite cardiac surgery.

**Results:**

A total of 341 centers were identified and 189/341 (55%) returned data sets were analyzed. Most types of EP procedures are performed across all kinds of centers. Ablation of ventricular tachycardia (VT) is concentrated in higher volume centers and in centers with onsite cardiac surgery. None of the participating low‐volume centers and only 13% of centers without onsite cardiac surgery responded to performing epicardial VT ablation. Irrespective of center volume and onsite cardiac surgery, neither body mass index nor age was reported to be an exclusion criterion for ablation procedures. Higher volume centers and centers with onsite cardiac surgery more often have dedicated EP laboratories and EP‐nursing teams. Also, differences regarding periprocedural safety precautions and management of cardiac tamponade were found for low‐, mid‐, and high‐volume centers, as well as for centers with and without onsite cardiac surgery.

**Conclusion:**

While center volume and onsite cardiac surgery do not impact patient selection, there are differences in ablation spectrum, infrastructure, periprocedural safety precautions, and treatment of tamponade.

## Graphical abstract

Results from this physician‐based national survey demonstrate that neither center volume nor onsite cardiac surgery impacts the ablation spectrum or patient selection during electrophysiological procedures, whereas there are important differences with regard to the infrastructure and treatment of pericardial tamponade.

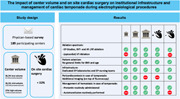

## 1 INTRODUCTION

Catheter ablation has become an established treatment option for a variety of cardiac arrhythmias. Nevertheless, periprocedural complications may occur and potentially result in life‐threatening conditions.^1^ Cardiac tamponade, in general, is one of the most feared complications in interventional electrophysiology (EP) and the most common major complication during atrial fibrillation (AF) ablation.^2^, ^3^ Previous studies focused on risk factors such as age and comorbidities and on predictors such as technologies used, as well as on overall and procedure‐specific outcomes.^1^, ^4^

However, the number of ablation centers, especially low‐volume centers without cardiac surgery, and contemporaneous evaluation of the number of more complex procedures have increased. Thus, the management of cardiac tamponade is still not standardized, and therefore various uncertainties, for example, on epicardial puncture technique, reversal of heparin effects, the use of autotransfusion, and the timing of involvement of cardiac surgery in severe cases, as well as subsequent monitoring and medication, still remain.

This analysis sought to evaluate the institutional infrastructure and the impact of center volume and onsite cardiac surgery on the acute management of cardiac tamponade and subsequent therapy during electrophysiological procedures. The underlying survey interrogated the management of cardiac tamponade in German ablation centers via a standardized questionnaire including queries on precautions and peri‐ and postprocedural management of cardiac tamponade.^5^

## 2 METHODS

The underlying physician‐based survey was conducted on May 2020 by sending out postal questionnaires to a total of 341 identified hospitals according to an official list containing all German centers performing EP procedures. The questionnaire, consisting of 46 questions, is provided in the Supporting information: Material. The postal questionnaires were sent to all identified EP centers, followed by up to two reminders. In case a center did not answer within 3 months, up to two reminders were given out. The results were obtained anonymously. All centers were asked to complete all questions. Since it was a paper‐based questionnaire, this was not mandatory. Ethics approval for the current anonymized survey was not necessary according to institutional ethical standards. The general results of this survey have been previously published.^5^

For this substudy, the results of the survey were analyzed for low‐volume (0–250 procedures per year), mid‐volume (250–500 procedures), and high‐volume (>500 procedures) centers, as well as for centers with and without onsite cardiac surgery, to evaluate the infrastructure of German EP centers and the impact of center volume and onsite cardiac surgery on the management of cardiac tamponade and subsequent therapy

The results are displayed as categorical data and described with absolute and relative frequencies. All calculations were performed with R version 3.6.0 (2019).

## 3 RESULTS

### 3.1 Baseline data

A total of 341 German ablation centers were identified and 189/341 (55%) data sets from all responding centers were included in our analysis.

Of 189 participating centers, 69 (36.5%) perform 0–250 EP procedures per year, 69 (36.5%) perform 250–500 procedures per year, and 51 (27%) perform >500 procedures per year.

The survey revealed that in total 61/189 (32%) EP centers have onsite cardiac surgery, whereas 128/189 (68%) centers have not.

It is worth noting that only 4/69 (6%) of all participating low‐volume centers and 22/69 (32%) of mid‐volume centers stated to have onsite cardiac surgery, while the majority of the high‐volume centers (34/51 (67%)) stated to have onsite cardiac surgery. All centers without onsite cardiac surgery are stated to collaborate with external cardiac surgery institutions.

### 3.2 Ablation spectrum

This subanalysis revealed that, irrespective of center volume, the large majority of all participating centers perform diagnostic EP studies and ablation of supraventricular tachycardia (SVT), as well as atrial flutter and AF ablation. AF ablation is the most commonly performed procedure in low‐volume (41%), mid‐volume (41%), and high‐volume (46%) centers, as well as at centers with (42%) and without (43%) onsite cardiac surgery. However, ablation of ventricular tachycardia (VT), especially epicardial VT ablation, is mainly carried out at high‐volume centers and centers with onsite cardiac surgery.

Left atrial appendage (LAA) closure is performed at 50/69 (72%) low‐volume centers, at 62/69 (90%) mid‐volume centers, and at 51/51 (100%) high‐volume centers. Also, 103/128 (81%) EP centers without onsite cardiac surgery reported to performing LAA closure.

**Figure 1:**
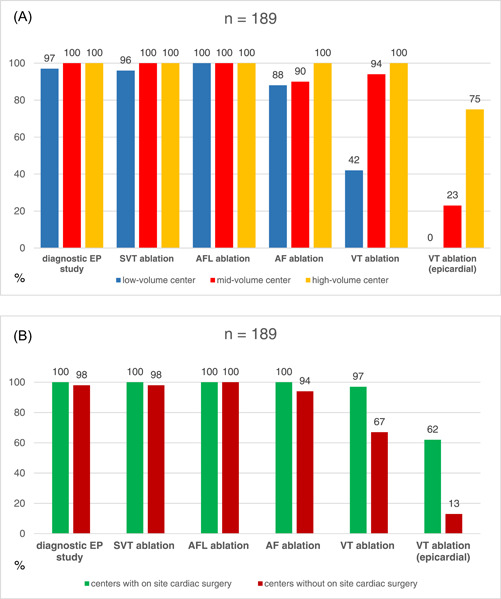
Percentagewise distribution of procedure type in low‐, mid‐, and high‐volume electrophysiology (EP) centers (A), as well as at centers with or without cardiac surgery (B). AFL, atrial flutter; SVT, supraventricular tachycardia; VT, ventricular tachycardia.

Details on ablation spectra at low‐, mid‐, and high‐volume centers, as well as centers with or without onsite cardiac surgery, are given in Figure 1.

### 3.3 Infrastructure

A total of 30/69 (43%) low‐volume, 53/69 (77%) mid‐volume, and 50/51 (98%) high‐volume centers have dedicated EP laboratories. Also, the number of centers with dedicated EP‐nursing teams rises with center volume (28% for low‐volume, 42% for mid‐volume, and 76% for high‐volume centers). Also, significantly more centers with onsite cardiac surgery (57/61, 93%) when compared to centers without onsite cardiac surgery (76/128, 60%) reported to have dedicated EP laboratories. Furthermore, 37/61 (60%) centers with onsite cardiac surgery, and only 45/128 (35%) of participating centers without onsite cardiac surgery have dedicated EP‐nursing teams.

**Figure 2:**
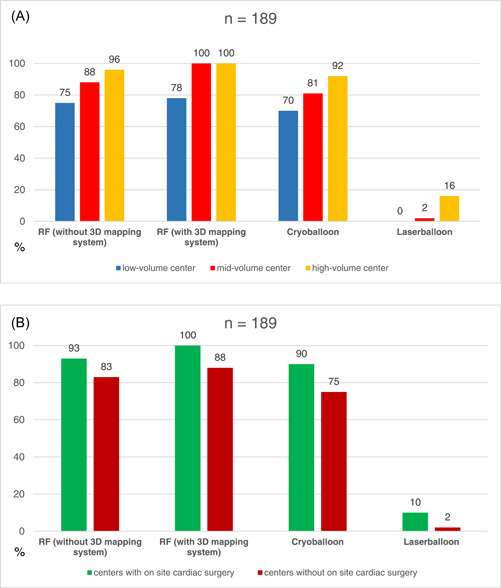
Percentagewise distribution of electrophysiology (EP) mapping and ablation tools in low‐, mid‐, and high‐volume EP centers (A). Percentagewise distribution of EP mapping and ablation tools at EP centers with and without onsite cardiac surgery (B). RF, radiofrequency.

The technical infrastructure in view of EP mapping and ablation platforms varies between low, mid‐, and high‐volume centers (Figure 2A). Interestingly, radiofrequency (RF) current in combination with or without a three‐dimensional (3D) mapping system is still the most commonly available and applied ablation strategy, irrespective of center volume.

Centers without onsite cardiac surgery also do not use balloon‐based ablation tools more often when compared to RF current with or without 3D mapping systems (Figure 2B).

### 3.4 Patient selection

In most of the low‐volume (59%), mid‐volume (86%), and high‐volume (65%) centers, as well as centers with (74%) and without (69%) onsite cardiac surgery, the body mass index (BMI) does not serve as a general exclusion criterion for EP procedures. However, irrespective of center volume and onsite cardiac surgery, most institutions have BMI limits only for left atrial and/or ventricular procedures.

Age as a general exclusion criterion for EP procedures was reported by only 2/69 (3%) low‐volume, 1/69 (1%) mid‐volume, and 0/51 (0%) high‐volume centers. None of the participating centers with onsite cardiac surgery and only 3/128 (2%) without onsite cardiac surgery reported age as a general exclusion criterion. Furthermore, only 17/69 (25%) of the participating low‐volume, 9/69 (13%) of all mid‐volume, and 6/51 (11%) of the high‐volume centers, as well as 25/128 (20%) of centers without and 7/61 (12%) of centers with onsite cardiac surgery, reported age limits only for left atrial and/or ventricular procedures.

### 3.5 Periprocedural safety

**Figure 3:**
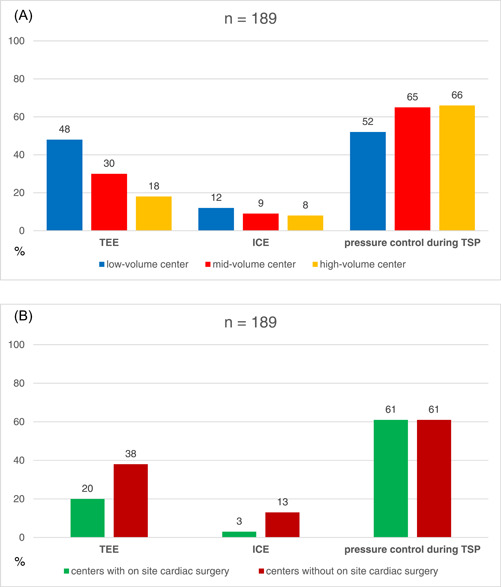
An overview of imaging and monitoring modalities additionally used for transseptal puncture by low‐, mid‐, and high‐volume electrophysiology (EP) centers (A), as well as centers with or without onsite cardiac surgery (B). ICE, intracardiac echocardiogram; TEE, transesophageal echocardiography; TSP, transseptal puncture.

A total of 30/69 (44%) low‐volume, 41/69 (59%) mid‐volume, and 38/51 (75%) high‐volume centers use fluoroscopy alone to guide transseptal puncture. In 65/128 (51%) centers without and 45/61 (74%) of centers with onsite cardiac surgery, transseptal puncture was performed without any additional imaging modality apart from fluoroscopy. An overview of additional imaging and monitoring modalities used for transseptal puncture by low‐, mid‐, and high‐volume EP centers, as well as at centers with or without onsite cardiac surgery, is provided in Figure 3.

In 93/189 (49%) and 139/189 (74%) centers, AF and VT ablations were performed under continuous invasive blood pressure measurement with significant differences depending on center volume. In 36/69 (52%) of the participating low‐volume, 40/69 (58%) of the mid‐volume, but only 17/51 (33%) of the high‐volume EP centers invasive blood pressure management was demonstrated during AF ablation procedures. During VT ablation, only 27/69 (39%) of the participating low‐volume, but almost all mid‐volume (63/69, 91%) and high‐volume centers (49/51, 96%) perform invasive blood pressure monitoring. A majority of centers without onsite cardiac surgery perform invasive blood pressure management during AF ablation (71/128, 55%), whereas EP centers with onsite cardiac surgery often refrain from doing so. During VT ablation, however, invasive blood pressure monitoring is used more frequently in centers with when compared to centers without onsite cardiac surgery (93% vs. 62%).

The majority of participating EP centers (180/189, 95%) have echocardiography permanently available onsite in the EP laboratory. Furthermore, almost all centers (187/189, 98%) have a dedicated pericardiocentesis set prepared for emergencies in the cath lab. In these regards, there are no relevant differences to report for low‐, mid‐, and high‐volume centers or EP centers with and without onsite cardiac surgery.

In general, 93/189 (49%) centers have regular trainings with the EP team to prepare for emergency intervention in case of cardiac tamponade, although this is more likely to be the case at high‐volume centers (71%) when compared to mid‐volume (46%) and low‐volume centers (36%). Also, slightly more centers with onsite cardiac surgery (59%) have special emergency trainings with the EP team when compared to centers without onsite cardiac surgery (45%).

### 3.6 Acute management of cardiac tamponade

Whenever pericardial tamponade occurs, 49/189 (26%) centers immediately contact an institutional resuscitation team (33% of all low‐volume, 17% of all mid‐volume, and 29% of all high‐volume centers). Another 25/189 (13%) centers always inform a cardiac surgeon in case of pericardial tamponade (10% of all low‐volume, 15% of all mid‐volume, and 16% of all high‐volume centers). Of note, twice as many centers with onsite cardiac surgery do always inform a surgeon in case of pericardial tamponade when compared to those without onsite cardiac surgery (21% vs. 9%).

Fluoroscopy as the primary and only imaging modality for pericardiocentesis is used in 53/189 (28%) of all participating institutions, and there are no differences to report for low‐, mid‐, and high‐volume centers or centers with and without onsite cardiac surgery. However, echocardiography is more often used for pericardiocentesis—in addition to fluoroscopy—at low‐volume centers when compared to mid‐ and high‐volume centers (84% vs. 63%), and at centers without when compared to those with onsite cardiac surgery (75% vs. 61%).

When cardiac tamponade occurs, protamine is routinely administered in most of all low‐volume (58/69, 84%), mid‐volume (60/69, 87%), and high‐volume (45/51, 88%) EP centers, and 56/61 (92%) of centers with and 107/128 (84%) without onsite cardiac surgery. Higher volume centers and centers with onsite cardiac surgery more often administer protamine once all blood is aspirated from the pericardial space, whereas low‐volume centers and centers without cardiac surgery administer protamine immediately when cardiac tamponade is diagnosed.

Only 6/69 (9%) of all low‐volume, 6/69 (9%) of the mid‐volume, and 5/51 (10%) of the high‐volume centers routinely apply a specific new oral anticoagulant antidote for adjunct treatment of cardiac tamponade. In this regard, there is also no difference to report for EP centers with when compared to those without onsite cardiac surgery (10% vs. 9%).

A total of 56/189 (30%) centers routinely administer clotting factors (prothrombin complex concentrate [PPSB], aPPSB, recombinant FVIIa); this is twice as common for low‐volume (35%) and mid‐volume (36%) when compared to high‐volume centers (14%). In this regard, there is no difference between centers with and without onsite cardiac surgery (26% vs. 29%).

With regard to autotransfusion of aspirated blood, 9/69 (13%) of the participating low‐volume, 17/69 (25%) of mid‐volume, and 10/51 (20%) of high‐volume centers responded that they reinfuse blood only before protamine administration, while 5/69 (7%) of all low‐volume, 11/69 (16%) of the mid‐volume, and 15/51 (29%) of the high‐volume centers autotransfuse after protamine administration. However, most of all participating low‐volume centers (47/69, 68%) and almost half of mid‐volume centers (34/69, 49%) do not perform autotransfusion, while only a minority of all participating high‐volume centers (20/51, 39%) answered to not perform autotransfusion at all. A majority of all participating centers without onsite cardiac surgery (74/128, (58%)), but only 27/61 (44%) of centers with onsite cardiac surgery denied performing autotransfusion. Fifteen out of 189 (10%) centers report other approaches. Three centers (2%) did not answer.

Most of the low‐volume (45/69, 65%), mid‐volume (45/69, 65%), and high‐volume (28/51, 55%) EP centers, as well as EP centers with (33/61, 54%) and without (85/128, 66%) onsite cardiac surgery, chose surgical treatment if the bleeding did not stop after all conventional treatment options. Fifteen out of 69 (22%) of the low‐volume, 10/69 (15%) of the mid‐volume, and 9/51 (18%) of the high‐volume centers, as well as 12/61 (20%) of centers with and 22/128 (17%) centers without onsite cardiac surgery, chose surgical treatment after a certain amount of blood was aspirated and if bleeding continued. The remaining centers have a different approach, which was not further specified.

### 3.7 Monitoring and subsequent therapy

Most of all participating centers monitor their patients in an intensive care unit (ICU) once pericardial tamponade is successfully treated, irrespective of center volume (low‐volume: 81%; mid‐volume: 80%; high‐volume: 78%) and onsite cardiac surgery (with onsite cardiac surgery: 77%; without onsite cardiac surgery: 81%).

**Figure 4:**
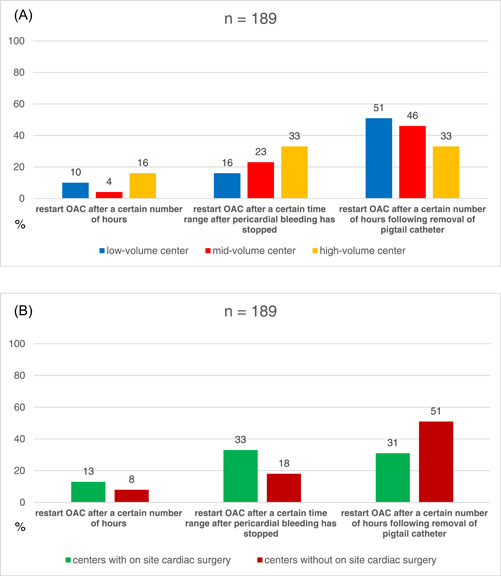
An overview of differences for low‐, mid‐, and high‐volume electrophysiology (EP) centers (A), and centers with and without cardiac surgery (B) regarding the restart of oral anticoagulant (OAC) after pericardiocentesis.

Thirteen out of 69 (19%) of the low‐volume, 26/69 (38%) of the mid‐volume, and 22/51 (43%) of the high‐volume centers, as well as 24/61 (49%) of the centers with and 36/128 (30%) of the centers without onsite cardiac surgery, stated that they routinely apply nonsteroidal anti‐inflammatory drugs (NSAIDs), colchicine, or cortisone after pericardial tamponade. Figure 4 gives details on the center's strategies for oral anticoagulant (OAC) restart after pericardiocentesis.

## 4 DISCUSSION

Evidence on the best management of pericardial tamponade during EP procedures is lacking. Nevertheless, most German EP centers have institutional standards, which were recently interrogated during a national survey on the management of cardiac tamponade in German EP centers via a standardized postal questionnaire including queries on infrastructure, precautions, periprocedural safety, acute management of cardiac tamponade, and subsequent therapy.^5^

This subanalysis reveals further interesting insights on the impact of ablation volume of centers and onsite cardiac surgery on the ablation spectrum, patient selection, technical and personnel infrastructure, periprocedural safety conditions, acute management of cardiac tamponade, and subsequent therapy.

The main findings of the underlying analysis are as follows:

1. Whereas VT ablation, and especially epicardial VT ablation, is mostly performed at high‐volume centers and centers with onsite cardiac surgery, no differences were observed for the spectrum of other ablation procedures.
2. Irrespective of center volume and onsite cardiac surgery, neither BMI nor age was reported to be a general exclusion criterion for ablation procedures.
3. High‐volume centers and centers with onsite cardiac surgery more often have dedicated EP laboratories and dedicated EP‐nursing teams.
4. In case of cardiac tamponade, protamine is routinely administered, while autotransfusion in general is more often performed at high‐volume centers and centers with onsite cardiac surgery.
5. High‐volume centers and centers with onsite cardiac surgery more often routinely apply NSAIDs, colchicine, or cortisone after pericardial tamponade.

### 4.1 Ablation spectrum and platforms

This subanalysis demonstrates that center volume and onsite cardiac surgery do not impact the ablation spectrum in general. The only exception to report is, however, that VT ablation, in particular with epicardial access, is mainly performed in high‐volume centers and centers with onsite cardiac surgery.

The recent 2019 HRS/EHRA/APHRS/LAHRS consensus document of VT ablation recommends onsite surgical backup for the conduction of epicardial VT procedures. An epicardial access and ablation bear various risks such as injury to adjacent organs like the liver, colon, or coronary arteries, puncture or laceration of the right ventricle as well as of the aorta, which might necessitate urgent surgical repair.^6^, ^7^ In 2020, Fink et al.^1^ retrospectively analyzed 34 982 consecutive patients undergoing diagnostic EP studies and/or catheter ablation of arrhythmias to identify predictors for tamponades with severe course. In their study, the frequency of tamponade was mainly dependent on the type of procedure that was performed and was highest in patients undergoing VT ablation with epicardial access (9.4%). Furthermore, among others, endocardial VT ablation and procedures requiring epicardial approach were found to be independent predictors of severe tamponade (e.g., requiring surgical repair, associated with periprocedural death). In this analysis, the necessity for surgical repair was >12% of patients with cardiac tamponade, in >10% of patients undergoing LA procedures, and >21% in patients undergoing endocardial or epicardial VT ablation,^1^ which underlines the need for an in‐house surgical backup during complex endocardial and epicardial VT ablation and support the current strategy in German EP centers.

The survey furthermore demonstrated that, irrespective of center volume and onsite cardiac surgery, AF ablation is the most commonly performed procedure (~40% of all procedures). In addition, within this survey, most centers also reported RF current in combination with or without 3D mapping system to be the most widely applied ablation strategy. One might therefore conclude that even low‐volume centers and centers without onsite cardiac surgery more often perform RF‐based AF ablation than balloon‐based AF ablation, although there is existing data demonstrating reduction of cardiac tamponade frequency when using balloon devices.^4^ This might be due to the fact that RF‐based ablation is still the most established ablation strategy with the greatest wealth of experience. However, it remains unclear whether the reported AF ablation procedures were first‐do procedures, which might have been suitable for balloon‐based ablation, or re‐do procedures, which are usually conducted using RF, as more complex ablation strategies might have been necessary in these cases.

### 4.2 Intrastructure

This subanalysis revealed that high‐volume centers and centers with onsite cardiac surgery more often have dedicated EP laboratories and dedicated EP‐nursing teams. In addition, these centers also more often reported to performing regular trainings with the EP team to prepare for emergency intervention in case of cardiac tamponade. A potential benefit of a more specialized infrastructure and purposefully trained staff might be an improvement not only in processes but also in the context of complications. However, as groups were divided according to center volume, and therefore overall caseload, the impact of individual operator's experience on the clinical outcome cannot finally be judged.

### 4.3 Patient selection

One of the most important steps to prevent major complications during catheter ablation is patient selection, and higher BMI, age, and international normalized ratio levels are considered as risk factors by physicians worldwide.

Most of the low‐, mid‐, and high‐volume centers and centers with and without onsite cardiac surgery reported that BMI did not serve as a general exclusion criterion for any kind of EP procedure. Nevertheless, irrespective of center volume and onsite cardiac surgery, still most institutions have BMI limits for left atrial and/or ventricular procedures. Although the prevalence of obesity is increasing, data on periprocedural complication rates of catheter ablation for arrhythmias are scarce. Recently, Schenker et al.^8^ published that obesity did not have a significant impact on the incidence of periprocedural complications after catheter ablation. They retrospectively analyzed 1000 consecutive patients undergoing catheter ablation of arrhythmias and found a major complication rate of 3.1% without a significant impact of BMI on the rate of major adverse events.^8^ However, radiation exposure and procedure duration were shown to be increased in obese patients,^8^ and ablation outcomes might be worse when compared to patients with normal weight.^9^ However, the improvement in quality of life might be consistent across all BMIs.^10^

Importantly, although several reports have shown that catheter ablation can be safely performed in the elderly,^11^, ^12^, ^13^ many German EP centers discard catheter ablation in patients aged >75 years and nearly all centers having participated in this survey refuse to do left atrial/ventricular procedures in octogenarians.^5^ This subanalysis demonstrates that this applies to low‐, mid‐, and high‐volume centers, as well as centers with and without onsite cardiac surgery. Thus, although various data exist with regard to age and its association with peri‐interventional risk, more robust data is obviously needed to enhance validity.

### 4.4 Autotransfusion

Autotransfusion might be of particular importance in massive bleeding caused by steam pop or perforation requiring continuous aspiration of blood for a longer time period and can prevent the necessity of foreign blood transfusion and associated risks such as allergies or severe infection, and might in rare situations enable for completion of the ablation procedure.^14^, ^15^, ^16^ However, there is no evidence in favor of the superiority of autotransfusion, and no general recommendation for autotransfusion or the timepoint for autotransfusion during the management of cardiac tamponade. Furthermore, direct retransfusion of pericardial blood carries the risks of hemolysis or thromboembolism.

However, a majority of all participating high‐volume centers (61%), half of all mid‐volume centers (51%), but only the minority of low‐volume centers (32%), answered to performing autotransfusion in case of cardiac tamponade during EP procedures. Most of all participating centers without onsite cardiac surgery (74/128, (58%)), whereas only 27/61 (44%) of centers with onsite cardiac surgery denied to performing autotransfusion.

Further randomized trials are needed to assess the benefit of autotransfusion in the management of cardiac tamponade.

## 5 LIMITATIONS

The underlying survey was only conducted in Germany with a response of roughly 50% of the addressed EP centers. Of note, any survey is limited by recording perceptions and not prospective raw data. Nevertheless, the underlying analysis provides first insights into the differences in infrastructure and management of cardiac tamponade depending on center volume and onsite cardiac surgery, However, further data are needed to reveal potential impact on clinical outcome.

## 6 CONCLUSION

Center volume and onsite cardiac surgery did not impact patient selection. However, institutional infrastructure, periprocedural safety precautions, and the acute management of cardiac tamponade are still inhomogeneous, especially with regard to the conduction of pericardiocentesis, handling of hemostasis, and subsequent therapy.

## CONFLICTS OF INTEREST STATEMENT

Laura Rottner received travel grants from EPD Solutions/Philips (KODEX‐EPD). Andreas Metzner received speaker's honoraria and travel grants from Medtronic, Biosense Webster, Bayer, Boehringer Ingelheim, EPD Solutions/Philips (KODEX‐EPD), and Cardiofocus. Andreas Rillig received travel grants from Biosense, Medtronic, St. Jude Medical, Cardiofocus, EP Solutions, Ablamap, and EPD Solutions/Philips (KODEX‐EPD) and lecture and consultant fees from St. Jude Medical, Medtronic, Biosense, Cardiofocus, Novartis, and Boehringer Ingelheim. Bruno Reißmann received speaker's honoraria and travel grants from Medtronic. Jan‐Per Wenzel received funding from the German Foundation of Heart Research (F/29/19) unrelated to the project and travel grants from Boston Scientific unrelated. Daniel Steven received speaker's honoraria and travel grants from Medtronic, Biosense Webster, Bayer, and Boston Scientific. Philipp Sommer received financial support for advisory board activities from Abbott, Biosense Webster, Boston Scientific, and Medtronic (no personal compensation). Paulus Kirchhof received research support for basic, translational, and clinical research projects from European Union, British Heart Foundation, Leducq Foundation, Medical Research Council (UK), and German Centre for Cardiovascular Research, from several drug and device companies active in AF, and has received honoraria from several such companies in the past, but not in the last three years. He is listed as an inventor on two patents held by the University of Birmingham (Atrial Fibrillation Therapy WO 2015140571 and Markers for Atrial Fibrillation WO 2016012783).

## Supporting information

Supporting information.

## Data Availability

The data that support the findings of this study are available from the corresponding author upon reasonable request.
