# Supplementary material for: The infrastructure of electrophysiology centers impacts the management of cardiac tamponade—Results from a national survey
Source: Clin Cardiol. 2023 Aug 1;46(10):1210–9. doi: 10.1002/clc.24096 (PMC10577558; doi:10.1002/clc.24096)
Supplement: Supplementary file 1 — Supporting information. [file CLC-46-1210-s001.pdf]

Universitäres
Herz- und Gefäßzentrum
UKE Hamburg

BASIS-INFORMATION

Ablationsspektrum - Infrastruktur - Ausstattung

1. Welche elektrophysiologischen Untersuchungen bzw. Ablationen werden in Ihrem Zentrum durchgeführt?

☐ diagnostische elektrophysiologische Untersuchung

☐ SVT-Ablation

☐ Vorhofflimmer-Ablation

☐ Vorhofflatter-/AT-Ablation

☐ VT-Ablation

☐ Epikardiale VT-Ablation

☐ LAA-Verschluss

2. Wie viele elektrophysiologische Eingriffe werden in Ihrem Zentrum insgesamt pro Jahr durchgeführt?

☐ < 100

☐ 100-250

☐ 250-500

☐ 500-1000

☐ 1000-2000

☐ >2000

3. Haben Sie mindestens ein Labor, das ausschließlich für EPUs/Ablationen genutzt wird?

☐ Ja

☐ Nein

4. Wie verteilen sich die Eingriffe prozentual?

diagnostische elektrophysiologische Untersuchung ______ %

SVT-Ablation ______ %

Vorhofflimmer-Ablation ______ %

Vorhofflatter-/AT-Ablation ______ %

VT-Ablation ______ %

Epikardiale VT-Ablation ______ %

LAA-Verschluss ______ %

5. Wie viele Untersucher führen in Ihrem Zentrum regelhaft elektrophysiologische Untersuchungen und Ablationen durch?

Insgesamt: ______

Davon

zertifizierte Elektrophysiologen (OÄ/FÄ) ______

OÄ / FÄ, nicht zertifiziert ______

Assistenzärzte ______

Fellows ______

6. Welche Ablationssysteme werden in Ihrem Zentrum eingesetzt?

☐ Hochfrequenzstrom konventionell

☐ Hochfrequenzstrom und 3D-Mapping

☐ Cryoballon

☐ Laserballon

☐ andere: ____

7. Gibt es an Ihrem Zentrum Pflegepersonal oder ein Pflegeteam, das ausschließlich für elektrophysiologische Eingriffe zuständig ist?

☐ Ja

☐ Nein

8. Haben Sie generelle Regeln, Ablationen nicht anzubieten aufgrund von

BMI:

☐ Ja, aber nur für linksatriale/-ventrikuläre Prozeduren

BMI-Grenze ______

☐ Ja, für alle Prozeduren

BMI-Grenze ______

☐ Nein

Lebensalter:

☐ Ja, nur für linksatriale/-ventrikuläre Prozeduren

Altersgrenze: ______

☐ Ja, für alle Prozeduren

Altersgrenze: ______

☐ Nein

für den INR: ______ Grenzwert

9. Wird in Ihrem Zentrum eine bestehende Therapie mit NOAKs vor dem Eingriff pausiert?

☐ Ja

☐ am Tag vorher

☐ am Vorabend

☐ am Tag des Eingriffs

☐ anderes Vorgehen:____

☐ Nein

10. Im Falle eines präinterventionellen Pausierens der OAK: Wann nehmen Sie eine notwendige OAK nach Ablation im Normalfall wieder auf?

☐ sofort

☐ nach ______ Stunden

☐ am Folgetag

11. Nutzen Sie eine der folgenden zusätzlichen Bildgebungsmodalitäten bei der transseptalen Punktion?

☐ TEE

☐ ICE

☐ andere (welche: ____)

☐ keine

12. Welche diagnostischen Katheter platzieren Sie für die transseptale Punktion?

☐ CS-Katheter

☐ His-Katheter

☐ Pigtail/Draht in der Aorta

☐ keine

13. Führen Sie die transseptale Punktion unter Druckmessung durch?

☐ Ja

☐ Nein

14. Wann applizieren Sie Heparin?

☐ Vor der transseptalen Punktion gewichtsadaptiert

☐ Vor der transseptalen Punktion, aber nur ______ I.E. *(den Rest nach der transseptalen Punktion)*

☐ Nach der transseptalen Punktion gewichtsadaptiert

15. Welche Ziel-ACT haben Sie bei linksatrialen/linksventrikulären Prozeduren?

_______ sec.

16. Führen Sie vor der transseptalen Punktion eine ACT-Messung durch?

☐ Ja

☐ Nein

17. In welchem Intervall werden die ACT-Messungen durchgeführt?

Alle _______ min

18. Gibt es an Ihrem Zentrum eine Herzchirurgie?

☐ Ja

☐ Nein

19. Wenn an Ihrem Zentrum keine Herzchirurgie vorhanden ist, haben Sie eine Kooperation mit einer externen Herzchirurgie?

☐ Ja

☐ Nein

20. Bei welchen Untersuchungen führen Sie periprozedural invasive Blutdruckmessungen durch?

☐ diagnostische elektrophysiologische Untersuchung

☐ SVT-Ablation

☐ VHF-Ablation

☐ Vorhofflattern/AT

☐ VT-Ablation

☐ LAA-Verschluss

21. Bei rein nicht-invasiven RR-Messungen: In welchem Intervall wird gemessen?

alle _______ min

22. Haben Sie ein Echokardiographiegerät im EPU-Labor?

☐ Ja

☐ Nein

23. Der postinterventionelle Ausschluss eines Perikardergusses erfolgt standardmäßig:

☐ Auf dem Untersuchungstisch

☐ Im Aufwachraum

☐ Auf Station

☐ Am Folgetag

☐ Immer nochmalig am Entlassungstag

☐ Nur bei klinischem Verdacht

☐ Anderes Schema ____

24. Nach Ablation wird der Patient standardmäßig

☐ zunächst im Aufwachraum überwacht

Wenn ja, wie lange

☐ ≤ 1 Stunde

☐ insgesamt ______ Stunden

☐ direkt auf die Normalstation verlegt

☐ direkt auf die Monitor-Station verlegt

25. Haben Sie ein spezielles „Perikardpunktionsset" für Notfälle vorbereitet?

☐ Ja

☐ Nein

26. Finden in Ihrem Zentrum regelmäßige Trainings mit dem EP-Team für den Fall einer Perikardtamponade statt?

☐ Ja

☐ Nein

VORGEHENSWEISE BEIM AUFTRETEN EINER PERIKARDTAMPONADE

27. Ziehen Sie im Falle einer Perikardtamponade das hausinterne Reanimations-/Notfallteam hinzu?

☐ Ja

☐ Nein

☐ Nur im folgenden Fall:____

____

28. Informieren Sie im Falle einer Perikardtamponade grundsätzlich den Herzchirurgen?

☐ Ja

☐ Nein

☐ Nur im folgenden Fall:____

____

29. Wenn nicht schon vorhanden, legen Sie im Falle einer Perikardtamponade einen arteriellen Zugang zur invasiven Blutdruckmessung?

☐ Ja

☐ Nein

☐ Nur im folgenden Fall:____

____

30. Nutzen Sie zur Perikardpunktion ein Bildgebungsverfahren?

☐ Fluoroskopie

In welchen Ebenen?

☐ RAO

☐ LAO

☐ AP

☐ Echokardiographie

☐ Nein

31. Setzen Sie im Falle einer Perikardtamponade bei vorhergehender/fortgeführter NOAK-Therapie ein spezifisches Antidot ein?

☐ Ja

☐ Nein

☐ Nur im folgenden Fall:____

____

32. Legen Sie im Rahmen der Perikardpunktion eine Schleuse ins Perikard ein?

☐ Ja

Wenn ja, wie groß ist die Schleuse:

☐ 5F

☐ 6F

☐ 7F

☐ 8F

☐ andere: _______

☐ Nein

33. Legen Sie einen Pigtail-Katheter in das Perikard ein?

☐ Ja

Wenn ja, welche Größe?

☐ 5F

☐ 6F

☐ 7F

☐ andere: _______

☐ Nein

34. Geben Sie Protamin?

☐ Ja

Wenn ja, wann?

☐ Sobald die Perikardtamponade auffällt

☐ Nachdem ein sicherer Zugang zum Perikard vorhanden ist

☐ Nachdem das Perikard nach Aspiration komplett blutleer ist

☐ Andere: _______

☐ Nein

35. Wieviel Protamin geben Sie?

☐ Standardmäßig 3000 I.E.

☐ Standardmäßig 5000 I.E.

☐ Antagonisierung des zuvor verabreichten Heparins im Verhältnis 1:1

☐ In Abhängigkeit der zuletzt gemessenen ACT

☐ Andere: _______

36. Haben Sie im Falle einer Perikardtamponade unter NOAK-Therapie jemals ein spezifisches Antidot eingesetzt?

☐ Ja (aufgrund von: ____)

☐ Nein

37. Erfolgt die Gabe von Gerinnungsfaktoren (PPSB, aPPSB, Rekombinanter FVIIa)?

☐ Ja

☐ Nein

38. Reinfundieren Sie das aus dem Perikard aspirierte Blut?

☐ Ja, nur vor Protamingabe*

☐ Ja, auch nach Protamingabe*

*Wenn ja,

☐ ohne Filter

☐ mit Filter

☐ mit Cellsafer

☐ Nein

☐ Anderes Vorgehen: ____

____

39. Nehmen Sie im Falle einer Perikardtamponade regelhaft arterielle BGAs ab?

☐ Ja

Wenn ja, in welchem Intervall?

☐ 15min

☐ 30min

☐ 60min

☐ Anderes Intervall: ______

☐ Nein

40. Wie bzw. wann treffen Sie die Entscheidung zur kardiochirurgischen Versorgung nach Umsetzung aller konventionellen Maßnahmen?

☐ Bei weiter nachlaufendem Erguss nach einer Zeit von ____________________________________

☐ Nach einer Mindestmenge von ______ ml aspiriertem Blut und weiter nachlaufendem Erguss

☐ Anderes Vorgehen: ____

____

41. Wo wird Ihr Patient nach der Versorgung der Perikardtamponade überwacht?

☐ Intensivstation

☐ Intermediate Care Unit

☐ Normalstation

42. Wann entfernen Sie den Pigtailkatheter aus dem Perikard?

☐ Sofort

☐ Nach 8h

☐ Nach 12h

☐ Nach 24h

☐ Anderes Vorgehen: ____

43. Behandeln Sie Patienten nach Perikardtamponade regelhaft mit

☐ NSAR für ______Tage

☐ Colchicin für ______Tage

☐ Cortison

☐ Keines der Präparate

44. Werden Ihre Patienten bei liegendem Pigtail-Katheter mit einem Antibiotikum abgedeckt?

☐ ja, für _____Tage

Präparat: ___________________________________

☐ Nein

45. Wann setzen Sie eine ggf. indizierte Antikoagulationstherapie nach Tamponade in der Regel wieder an?

☐ nach ______ Stunden

☐ ______ Stunden nach Sistieren des Abflusses aus der Drainage

☐ ______ Stunden nach Entfernen der Drainage

☐ anderes Vorgehen: ____

46. Werden Patienten nach Perikardtamponade poststationär ambulant zur Verlaufskontrolle in Ihr Zentrum einbestellt?

☐ Ja

Wenn ja, nach wie vielen Tagen? ______

☐ Nein

Vielen Dank für Ihre Zeit und Mühe!
